# Colorectal Cancer Migration and Invasion Initiated by microRNA-106a

**DOI:** 10.1371/journal.pone.0043452

**Published:** 2012-08-17

**Authors:** Bo Feng, Tao Tao Dong, Lin Lin Wang, Hou Min Zhou, Hong Chao Zhao, Feng Dong, Min Hua Zheng

**Affiliations:** 1 Department of Surgery, Ruijin Hospital, Shanghai Jiaotong University School of Medicine, Shanghai, People’s Republic of China; 2 Shanghai Institute of Digestive Surgery, Shanghai, People’s Republic of China; 3 Shanghai Minimally Invasive Surgery Center, Shanghai, People’s Republic of China; 4 Department of Obstetrics and Gynecology, Qilu Hospital of Shandong University, Ji’nan, Shandong, People’s Republic of China; 5 Shanghai Institute of Medical Genetics, Shanghai Children’s Hospital, Shanghai Jiao Tong University School of Medicine, Shanghai, People’s Republic of China; University of Kentucky College of Medicine, United States of America

## Abstract

MicroRNAs have been implicated in the regulation of several cellular signaling pathways of colorectal cancer (CRC) cells. Although emerging evidence proves that microRNA (miR)-106a is expressed highly in primary tumor and stool samples of CRC patients; whether or not miR-106a mediates cancer metastasis is unknown. We show here that miR-106a is highly expressed in metastatic CRC cells, and regulates cancer cell migration and invasion positively *in vitro* and *in vivo*. These phenotypes do not involve confounding influences on cancer cell proliferation. MiR-106a inhibits the expression of *transforming growth factor-β receptor 2* (*TGFBR2*), leading to increased CRC cell migration and invasion. Importantly, miR-106a expression levels in primary CRCs are correlated with clinical cancer progression. These observations indicate that miR-106a inhibits the anti-metastatic target directly and results in CRC cell migration and invasion.

## Introduction

Metastases lead to approximately 90% of colorectal cancer (CRC)-related mortalities, yet the underlying mechanisms remain largely unclear [1]. Metastasis is a complex, multiple processes whereby CRC cells invade surrounding tissues, intravasate into the vasculature, translocate through the systemic circulation, extravasate into the parenchyma of distant tissues (liver and lungs), establish micrometastases, and form macroscopic secondary tumors [2]. In recent years, multiple studies have been conducted to investigate the genes and their products that are involved in metastasis [3]–[7].

MicroRNAs (miRRNs) comprise an evolutionarily-conserved class of small RNAs that can silence gene expression post-transcriptionally through sequence-specific interactions with the 3′ untranslated regions (UTRs) of cognate mRNA targets [8]. Recently, the role for miRNAs in the establishment and progression of CRC has become evident. Greater than 50% of miRNA genes are situated in fragile chromosomal regions that are altered during tumor progression [9], and some miRNAs have been identified as oncogenes or tumor suppressor genes in CRC [10]–[14]. In addition, expression profiling analysis has revealed characteristic miRNA signatures that can predict the clinical outcomes of CRC [15], [16].

miR-106a (miRBase: MIMAT0000103) has been shown to be highly expressed in cancer tissues and stool samples of CRC patients[16]–[18]; however, the precise role played by miR-106 in CRCs is unknown. We therefore correlated miR-106a with CRC migration and invasion, hoping that such an association might provide insight into the mechanisms by which CRC cells metastasize.

## Results

### miR-106a is Highly Expressed in Metastatic CRC Cells

**Figure 1:**
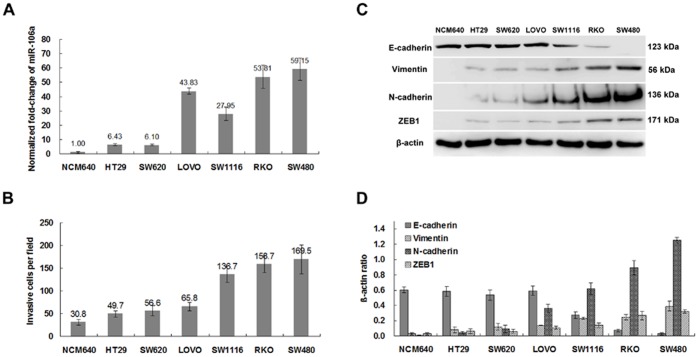
miR-106a is highly expressed in metastatic CRC cells. A, Real-time RT-PCR analysis of miR-106a expression in immortalized, normal human colon epithelial cells and a variety of CRC cells. U6 small nuclear RNA was used as an internal control. A representative experiment is shown in triplicate, and the bars indicate standard errors. B, Invasive ability of a series of CRC cells determined by Matrigel invasion assays. A representative experiment in triplicate is shown, along with standard errors. C, Western blot analysis of N-cadherin, E-cadherin, vimentin, and ZEB1 expression in CRC cells. D, quantitative analysis with image intensifier. *n* = 3, *bars,* ±SD.

We first determined the levels of miR-106a expression in a variety of human CRC cells. In agreement with previous reports [16]–[18], miR-106a was up-regulated in all six human CRC cell lines when compared to spontaneously immortalized, normal human colon epithelial cells (NCM640; Fig. 1A). miR-106a was more highly expressed in more invasive cells relative to cells with less invasive capacity (Fig. 1A and B). For example, the level of expression of miR-106a was 9-fold higher in the more invasive SW480 cell line than the less invasive HT29 cell line.

Furthermore, the expression of epithelial-mesenchymal transition (EMT) markers (E-cadherin, N-cadherin, vimentin, and ZEB1) was determined by Western blot analysis. EMT can be considered a pathologic process that contributes to cancer progression, particularly as it relates to invasion and metastasis. As shown in Fig. 1C and D, the levels of expression of E- and N-cadherin varied among seven cell lines. E-cadherin was expressed in NCM640 and five CRC cell lines, but not SW480, and more strongly expressed in NCM640, HT29, SW620, and LOVO. N-cadherin, vimentin, and ZEB1 expression was lower in the four cell lines.

The two CRC cell lines, RKO and SW480, which have the highest invasion potential, exhibited consistently low expression of E-cadherin and high expression of N-cadherin, vimentin, and ZEB1.

### miR-106a Initiates CRC Cell Migration and Invasion *in vitro*

**Figure 2:**
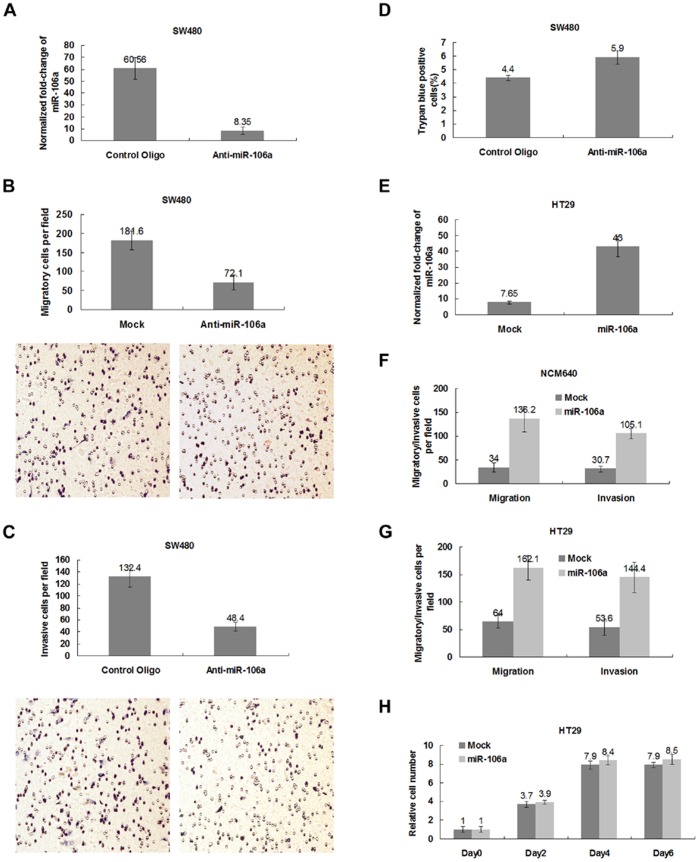
miR-106a initiates CRC cell migration and invasion *in vitro*. A, Levels of miR-106a expression in SW480 cells after transfection with inhibitors of miR-106a, as determined by real-time RT-PCR analysis. Data were normalized with U6 small nuclear RNA. A representative experiment in triplicate, along with standard errors is shown. B, Transwell migration assays of SW480 cells transfected with inhibitors of miR-106a or control vectors. A representative experiment is shown in triplicate and the bars denote standard errors. Also shown are phase-contrast images of SW480 cells migrating through membranes. C, Matrigel invasion assays of SW480 cells transfected with oligonucleotides against miR-106a or control oligonucleotides. A representative experiment in triplicate, as well as standard errors is shown. Phase-contrast images of SW480 cells invaded through membranes are also shown. D, Trypan blue exclusion assay of SW480 cells with miRNA inhibitors. A representative experiment is shown in triplicate, along with standard errors. E, Real-time RT-PCR analysis of miR-106a expression in HT29 cells infected with miR-106a-expressing or control vectors. U6 small nuclear RNA is used as an internal control. A representative experiment is shown in triplicate, and the bars indicate standard errors. F, Migration potential of NCM640 cells infected with miR-106a-expressing or control vectors, as determined by transwell migration assays. A representative experiment in triplicate, as well as standard errors is shown. Phase-contrast images of HT29 cells migrated through membranes are also shown. G, Invasive potential of HT29 cells after miR-106a transduction or control vector transduction measured by matrigel invasion assays. A representative experiment is shown in triplicate and the bars denote standard errors. Also shown are phase-contrast images of HT29 cells invaded through membranes. H, Growth curves of HT29 cells infected with the miR-106a-expressing or control vector *in vitro*. A representative experiment is shown in triplicate, along with standard errors.

We next undertook *in vitro* loss-of-function analyses to determine the role played by miR-106a in CRC cell migration and invasion. miR-106a was silenced *in vitro* via anti-sense oligonucleotides, then the levels of expression were determined by real-time RT-PCR [19]. We found that transfection of miR-106a anti-sense oligonucleotides in SW480 cells resulted in 7.3-fold lower levels of miR-106a expression (Fig. 2A). Anti-sense inhibitors of miR-106a led to a 2.5-fold decrease in the migration of SW480 cells, as determined by migration assays *in vitro*, relative to the control oligonucleotides (Fig. 2B). miR-106a inhibitors caused a 2.7-fold reduction in the invasive capacity of SW480 cells measured by invasion assays *in vitro* compared with control oligonucleotides (Fig. 2C). Importantly, this reduction could not be attributed to the destruction of cellular viability (Fig. 2D, P>0.05). Thus, these observations indicate that miR-106a is necessary for the migration and invasion of these more metastatic CRC cells *in vitro*, but not required for viability.

To determine whether or not overexpression of miR-106a conferred migratory and invasive potential on non-metastatic or less metastatic CRC cells, we cloned the genomic sequence of the human miR-106a gene into a mouse stem cell retrovirus-derived vector [20]. We then used the resulting vectors to express miR-106a in immortalized NCM640 and HT29 cells with less metastatic potential. Successful transductions of miR-106a were ascertained by real-time RT-PCR (Fig. 2E).

In both of these two lines, ectopic expression of miR-106a caused a significant increase in cellular migration and invasion (Fig. 2F and 2G). Such an increase cannot be due to the increased proliferative rates of these cells (Fig. 2H, P>0.05 for days 0, 2, 4, and 6). These observations suggest that overexpression of miR-106a is sufficient to initiate migration and invasion of CRC cells *in vitro*.

### miR-106a Promotes CRC Invasion *in vivo*

We next determined whether or not miR-106a induced invasion *in vivo*. To this end, we overexpressed miR-106a in CRC cells that are normally less invasive. First, we implanted HT29 cells transduced with miR-106a or infected with control vectors into nude mice subcutaneously. Recipient mice grew remarkable tumors within 2 weeks after injection, and became moribund at week 8 post-injection due to tumor burden when the experiment was terminated.

**Figure 3:**
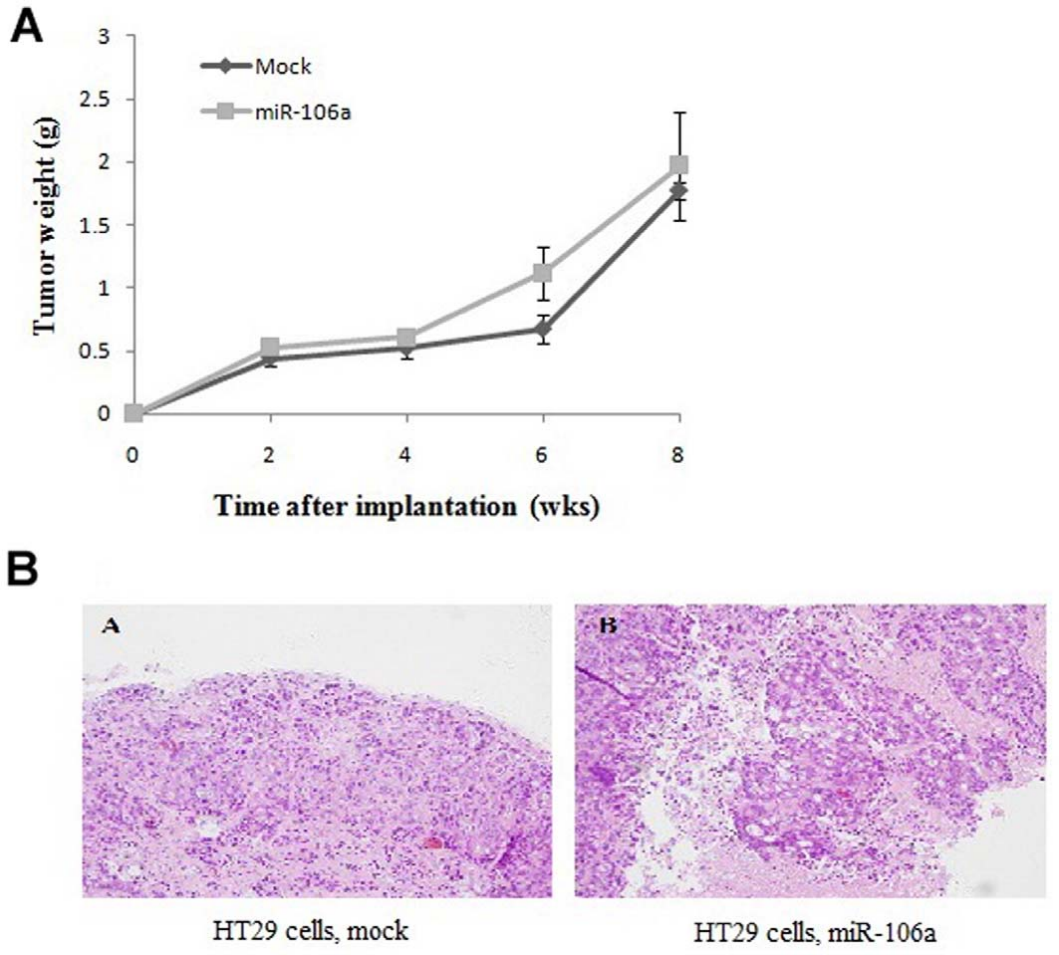
miR-106a promotes CRC invasion *in vivo*. A, Growth curves of tumors formed by HT29 cells infected with miR-106a-expressing or control vectors. Every data dot represents the mean ± standard error of 3–4 mice. B, Haematoxylin and eosin-stained sections of CRCs formed by HT29 cells infected with miR-106a-expressing or control vectors 8 weeks after implantation.

At week 4 after implantation, tumors of miR-106a-overexpressing HT29 cells and tumors of control infected cells were similar in size, suggesting that miR-106a had a minimal effect on primary tumor growth (Fig. 3A). As anticipated, tumors of control cells were non-invasive, shown as tumor masses confined within fibrous capsules (Fig. 3B, left panel). In contrast, tumors of miR-106a-overexpressing cells had islands of epithelial cancer cells invading into adjacent stromal tissues (Fig. 3B, left panel). Thus, ectopic expression of miR-106a conferred invasive capacity on cancer cells that are otherwise non-invasive.

### *TGFBR2* is a Direct Target of miR-106a

**Figure 4:**
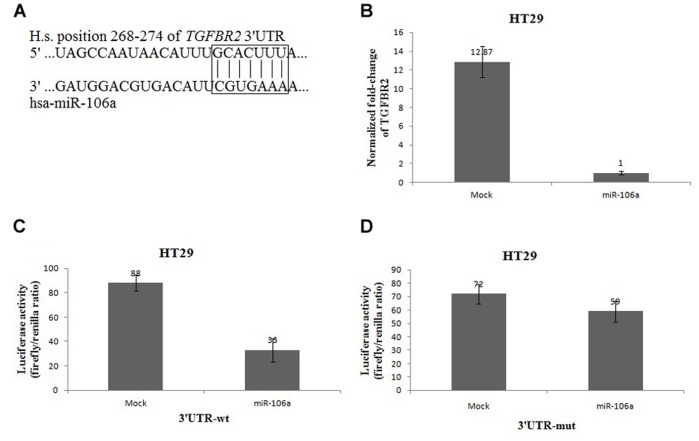
*TGFBR2* is a direct target of miR-106a. A, Predicted formation of duplex between human *TGFBR2* 3′UTR and miR-106a. B, Real-time RT-PCR analyses of *TGFBR2* in HT29 cells infected with miR-106a-expressing or control vector. *GAPDH* mRNA is used as an internal control. A representative experiment is shown in triplicate, along with standard errors. C, Luciferase activity of wild-type *TGFBR2* 3′UTR reporter gene in HT29 cells infected with miR-106a-expressing or control vectors. A representative experiment is shown in triplicate, along with standard errors. D, Luciferase activity of mutant-type *TGFBR2* 3′UTR reporter gene in HT29 cells infected with miR-106a-expressing or control vectors. A representative experiment is shown in triplicate. Bars denote standard errors.

To understand the mechanism by which miR-106a induce cancer cell migration and invasion, we used two algorithms (PicTar and TargetScan) to identify targets of human miR-106a [21], [22]. Among 990 targets predicted by TargetScan, the *LAMA3* (Gene ID: 3909) and *TGFBR2* genes (Gene ID: 7048) have previously been implicated in the regulation of cellular migration and/or invasion [23]–[27]. *TGFBR2* is particularly interesting because the expression of *TGFBR2* has been shown to be lost progressively during malignant progression of diverse types of cancer [23], [25], [27], [28]. In addition, TGFBR2-encoding mRNA has a 3′UTR element that is complementary to miR-106a (Fig. 4A).

Indeed, overexpression of miR-106a led to degradation of *TGFBR2* mRNA, as measured by real-time RT-PCR (Fig. 4B). We next determined whether or not *TGFBR2* is a direct target of miR-106a-mediated inhibition by assaying the activity of a luciferase reporter gene fused into the 3′UTR of the wild-type *TGFBR2* gene. miR-106a reduced the activity of the luciferase reporter significantly (P<0.05, Fig. 4C). Inhibition of *TGFBR2* mediated by miR-106a depends on the presence of miR-106a homologous binding sites in the 3′UTR of the *TGFBR2* gene. Because the luciferase reporter carries a mutant *TGFBR2* 3′UTR, substitution of 4 nucleotides within the miR-106a binding sites were not inhibited by miR-106a ectopic expression (Fig. 4D, P>0.05). Collectively, these observations indicate that *TGFBR2* may function as a direct target of miR-106a-mediated silencing.

### *TGFBR2* is a Functional Target of miR-106a

**Figure 5:**
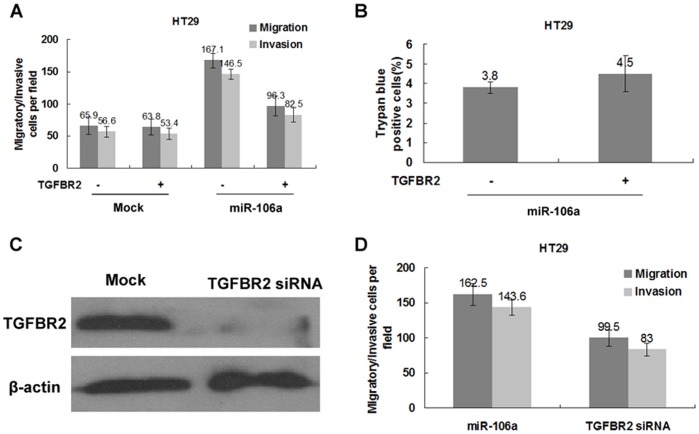
*TGFBR2* is a functional target of miR-106a. A, Matrigel invasion assays of miR-106a-transduced or control-infected HT29 cells transiently transfected with *TGFBR2*. A representative experiment is shown in triplicate along with standard errors. B, Trypan blue exclusion assay of miR-106a-expressing HT29 cells transduced *TGFBR2* or control vector. Shown is a representative experiment in triplicate, along with standard errors. C, Immunoblotting for *TGFBR2* in HT29 cells transiently transfected with *TGFBR2* siRNA. A representative experiment is shown in triplicate, along with standard errors. D, Comparison of the increased migration and invasion potential between HT29 cells transduced with miR-106a and HT29 cells transiently transfected with *β* siRNA. A representative experiment is shown in triplicate. Bars denote standard errors.

We next determined whether or not reduced expression of the *TGFBR2* gene is necessary for the induction of cellular migration and invasion observed after miR-106a overexpression. We overexpressed miR-106a and a construct expressing *TGFBR2* constitutively. This construct encodes the entire encoding sequence of *TGFBR2,* while lacking the 3′UTR element, thus yielding a type of mRNA resistant to miR-106a mediated inhibition. Remarkably, the resulting constitutively-expressed *TGFBR2* abrogated the previously elevated migration and invasion initiated by miR-106a (Fig. 5A), but had no effect on cell viability (Fig. 5B, P>0.05), suggesting that *TGFBR2* is indeed a functional target of miR-106a.

We also wanted to clarify whether or not degradation of *TGFBR2* is sufficient for the miR-106a-mediated increase of migration and invasion. Transfection of *TGFBR2* small interfering RNA (siRNA), which resulted in a >90% reduction in the levels of *TGFBR2* protein (Fig. 5C), led to a remarkable, but not complete induction of cell migration and invasion when compared to the induction caused by miR-106a overexpression (Fig. 5D). Collectively, *TGFBR2* appears to be a necessary, but not sufficient target of miR-106a.

### miR-106a Expression is Increased in Metastatic Primary CRC

**Figure 6:**
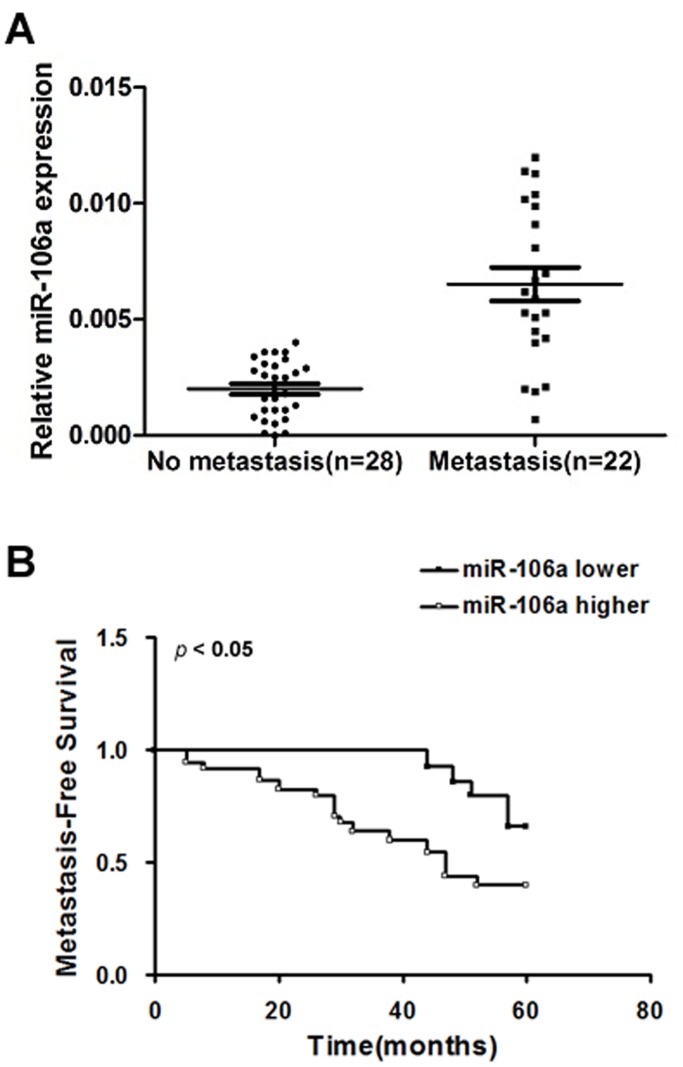
miR-106a expression is increased in metastatic primary CRCs. A, Levels of miR-106a expression in primary CRCs with or without metastasis. There was a significant difference in miR-106a expression in these two groups. B, Kaplan-Meier metastasis-free survival for 28 CRC patients without metastasis at diagnosis, stratified based on the levels of miR-106a expression in their primary tumors. Aχ^2^ test was used for statistical analysis and a P<0.05 was considered significant.

Importantly, a recent microarray analysis showed that miR-106a is among those miRNAs that are highly expressed in primary CRC when compared to normal colorectal epithelial tissue [16], [18]. We determined whether or not miR-106a expression in metastasis-positive tumors is higher than metastasis-free tumors, and whether or not miR-1061 expression is associated with clinical outcomes in CRC patients. We recruited 28 CRC patients who were long-term metastasis-free survivors after complete surgical resection of the primary tumor, and compared the patients to a control cohort of patients with metastasis at time of diagnosis or metastasis in follow-up (<60 months) (Table S1). Furthermore, the metastasis-free survival rates of patients without metastasis at diagnosis were stratified based on miR-106a expression status. Indeed, metastasis-positive patients had significantly higher levels of miR-106a in their primary tumors relative to the patients with no metastases (Fig. 6A, P<0.05). Notably, among all of these patients, those who had lower levels of miR-106a had better clinical outcomes (Fig. 6B, *p*<0.05). Collectively, these results indicate that miR-106a has an important role in CRC metastasis.

## Discussion

The present work identified miR-106a as a pro-metastasis miRNA leading to the promotion of migration and invasion of CRC cells. Reduction of this miRNA in CRC cells that are otherwise metastatic decreased the migratory and invasive potential of the CRC cells. In contrast, up-regulation of miR-106a in CRC cells with less migration and invasion potential increased cellular migration and invasion. We showed that miR-106a does not significantly influence the proliferative rates of CRC cells *in vitro* and *in vivo*. Such evidence suggests that miR-106a might only play a role in CRC cell invasion.

Interestingly, although from the same patient, SW480 and SW620 cells had different invasion potential and miR-106a expression (Fig. 1A and B). It is possible that this finding was because SW480 cells are from a primary cancer undergoing EMT and SW620 cells are from a secondary cancer. The decline in expression of ZEB1 in SW620 cells compared with SW480 cells may result in restoration of E-cadherin levels and induce a reverse process of EMT, or mesenchymal-epithelial transition (MET) [29], in which miR-106a may play a role as an upstream or downstream factor of ZEB1.

Our findings report for the first time that as a target gene of miR-106a, *TGFBR2* may be negatively regulated by miR-106a at the transcriptional level via binding of the 3′UTR of *TGFBR2* mRNA in CRC. *TGFBR2* is a major TGF-β signaling molecule often found to be one of the genes altered in cancer. The TGF-β signaling pathway plays a complex role, which remains controversial, in the malignant progression of tumors. It has been shown that TGF-β can induce the EMT and promote tumor cell invasion [30], whereas another study has shown that reduced *TGFBR2* expression is associated with aggressive features of hepatocellular carcinoma [25]. In CRC, TGF-β may induce growth inhibition of cancer cells, and *TGFBR2* was a tumor suppressor protein that is required for TGF-β signaling [31]. The present study showed that down-regulation of *TGFBR2* increases cellular migration and invasion of HT29 (Fig. 2G and 5D). Therefore, TGF-β signaling may be an important approach by which miRNAs regulate the development of tumors. A recent study reported that miR-17∼92, activated by c-Myc, attenuates the TGFβ signaling pathway, thereby stimulating angiogenesis and tumor cell growth [32]. Although *TGFBR2* appears to be a necessary, but not sufficient target of miR-106a in the current study, the relationship between miR-106a and TGF-β signaling deserves further study.

Clinicopathologic analysis further demonstrated the connection between miR-106a and metastasis. Long-term survivors without metastasis had significantly lower levels of miR-106a in their primary tumors relative to patients with disease recurrence, and lower levels of miR-106a suggests a higher metastasis-free survival rate (Fig. 6). This finding strongly supports the notion that overexpression of miR-106a is intimately involved in the metastasis of CRC.

In conclusion, we showed that the expression of miR-106a was positively correlated with the migration and invasion potential of CRC cells, and down-regulation of *TGFBR2*, as one of the target genes for miR-106a, could increase cell migration and invasion.

More importantly, the levels of miR-106a expression in primary CRCs are correlated with clinical cancer progression, which suggests that miR-106a may represent a novel target for therapeutic intervention to prevent CRC metastasis.

## Materials and Methods

### Cell Lines

The immortalized human colon epithelial cell line,NCM640, was a gift from J.Q. Zhang and cultured in DMEM supplemented with 10% FBS HT29, SW480, LOVO, SW1116, and SW480 cell lines were purchased from the ATCC (Manassas, VA, USA) and cultured under conditions in accordance with the manufacturer’s instructions.

### RNA Isolation and miRNAs Assays

Total RNA of cultured cells was extracted with mirVana Isolation Kit (AM1560, Ambion, Austin, TX, USA ). miRNAs assays were performed with mirVana qRT-PCR miRNA Detection Kit (AM1558, Ambion) together with qRT-PCT Primer Sets (4395280, Ambion) in accordance with manuals of manufactures. U6 small nuclear RNA was used as internal control.

### Oligonucleotide Transfection

The miRIDIAN microRNA inhibitor, miRIDIAN Mimic hsa-miR-106a and siRNA of *TGFBR2* was purchased from Dharmacon (IH-300526-08, C-300526-07, and D-003930, Dharmacon, Lafayette, CO, USA). Cells were transfected with 200 nM of the indicated oligonucleotides using Oligofectamine Reagent (12252011, Invitrogen, Carlsbad, CA, USA). Cells were used for subsequent experiments 48 h after transfection.

### Gene Cloning and Ectopic Expression

The human miRNA gene was PCR-amplified from normal genomic DNA and cloned into the MDH1-PGK-GFP 2.0 retrovirus-derived vector. *TGFBR2* cDNAs lacking the 3′UTR was PCR-amplified from normal genomic DNA and expressed from pWZL-blasticidin vector. Viral generation and infection of target cells have been described previously [33]. Infected cells were selected with 2 µg/ml of puromycin (P9620, Sigma, St. Louis, MO, USA), 200 µg/ml of hygromycin (H9773, Sigma), and 10 µg/ml of blasticidin (sc-205604, Santa Cruz, Santa Cruz, CA, USA).

### In vitro Migration and Invasion Assays

The migration and invasion potential of CRC cells was evaluated, as described previously with slight modifications [34].

CRC cells (2.5×10^5^) were plated on uncoated upper chambers (24-well inserts; pore size, 8 µm; BD Bioscience, Bedford, MA, USA) for transwell migration assays. CRC cells (2.5×10^5^) were placed on Matrigel-coated upper chambers (24-well inserts; pore size, 8µm; BD Bioscience) for invasion assays.

The medium in the top component was aspirated and replaced with serum-free media 2 h after seeding, and medium containing 20% serum was used as a chemotractant in the lower chambers. Cells were allowed to migrate for 24 h, and then the cells on both sides of the chamber were fixed with 50/50% acetone/methanol. Cells at the bottom of the chamber were stained with crystal violet (C3886, Sigma). Then, the picture of each well was taken to image the nuclei of the cells with the ×10 objective, and the cell nuclei in each field was counted using Image-J software (NIH; http://rsb.info.nih.gov/ij/).

### Animal Experiments

Six-to-eight week old nude mice were used for the study, and all the experimental procedures were approved by the Animal Care Committee of Shanghai Jiaotong University. 10^6^ HT29 cells in 200 µl DMEM were injected into the flanks of mice subcutaneously. The diameters of tumors were measured twice a week with precision calipers. Three-to-four mice per group were euthanized at each of the 4 time points (weeks 2, 4, 6, and 8 post-transplantation). The tumors were removed and weighed. Tissue samples were fixed in 10% buffered formalin for 12 h, then washed with PBS and submitted to 70% ethanol, followed by being embedded in paraffin, sectioned, and stained with haematoxylin and eosin (H9627 and E4009, Sigma).

### SYBR Green Real-time RT-PCR

The total RNA of cells was extracted and reverse-transcribed, as described previously [35]. The upstream and downstream primers for the *TGFBR2* gene were obtained from Primerbank (http://pga.mgh.harvard.edu/primerbank/): 5′-GTAGCTCTGATGAGTGCAATGAC-3′ and 5′-CAGATATGGCAACTCCCAGTG-3′. SYBR green real-time RT-PCR and the corresponding data analysis have been described previously [35]. β-actin mRNA were used as an internal control (primers : 5′-CATGTACGTTGCTATCCAGGC-3′ and 5′-CTCCTTAATGTCACGCACGAT-3′).

### Immunoblotting

Cells were harvested in RIPA lysis buffer. Proteins from cellular lysates were resolved with PAGE gel, transferred to PVDF membranes (Millipore Corp., Billerica, MA, USA), then blocked in 5% non-fat milk in PBS/Tween-20, followed by incubation with antibody againstN-cadherin (sc-271386, Santa Cruz), E-cadherin (sc-21791, Santa Cruz), vimentin (sc-373717, Santa Cruz), ZEB1 (sc-25388, Santa Cruz) or TGFBR2 (sc-17799, Santa Cruz). The quantity of target protein was calibrated using β-actin (sc-130656, Santa Cruz), and relative intensities were obtained.

### Construct

*TGFBR2* 3′UTR sequences were cloned into a pMIR-REPORT luciferase construct (AM5795, Ambion) [36]. Mutant *TGFBR2* 3′UTR was generated with a QuickChange Site-Directed Mutagenesis Kit (200519, Stratagene, Palo Alto, CA, USA).

### Luciferase Reporter Assay

Cells at 50% confluence in 24-well plates were transfected with Fugene6 (E2691, Promega, Madison, WI, USA). The luciferase reporter gene construct (200 ng) and pRL-SV40 Renilla luciferase construct (1 ng [used for normalization]) were co-transfected in each well. Cell extracts were prepared 24–48 h after transfection and measured with a Dual-Luciferase Reporter Assay System (E1910, Promega).

### Clinical Colorectal Tumors

Primary colorectal tumors and corresponding normal colorectal tissue were collected between 2004 and 2006, and processed in accordance with a protocol approved by the Ethical Committee of Ruijin Hospital of the Shanghai Jiaotong University School of Medicine. The TNM stage of the tumor was determined according to the classification proposed by the AJCC Cancer Staging Manual [37]. Follow-up data were summarized at the end of 2011 with a median follow-up of 60 months. Fresh tissue was harvested from patients, snap-frozen, and preserved at −80°C. Partial sections from the same tissues were then stained with haematoxylin-eiosin to validate the presence of tumor and the tissues containing >90% tumor cells were used for qRT-PCR as tumors. Total RNA was isolated from frozen tissue using TRIzol extraction (15596, Ambion) and a mirVana miRNA Isolation Kit (AM1560, Ambion). miRNA assays were performed with a mirVana qRT-PCR miRNA Detection Kit (AM1558, Ambion) together with qRT-PCT Primer Sets (4395280, Ambion) in accordance with the manufacturer’s manuals. U6 small nuclear RNA was used as an internal control. Primary tumors were stratified as miR-106a lower or higher to discern whether or not miR-106a levels correlated with metastasis. Tumors were considered miR-106a lower or higher if the normalized expression of miR-106a resided in the bottom or top 50% of the tumors in the group, respectively.

### Cell Growth and Viability Assay

To determine growth rates, 2.5×10^4^ cells were plated in each well of a 12-well plate. Twenty-four hours later, the cells were harvested and counted on a hemocytometer every 2 days until day 6. To determine cell viability, cells were trypsinized and diluted with 0.4% trypan blue (302643, Sigma) staining solution and counted on a hematocytometer.

### Statistical Analysis

Data are expressed as the mean ± standard errors. A Student t-test (two-tailed) was used to compare two groups (a P<0.05 was considered significant) unless indicated otherwise (χ^2^ test).

## Supporting Information

Table S1 **Correlation of miR-106a expression with metastasis in colorectal cancer patients.**
(DOC)
