# Correction: Colorectal Cancer Migration and Invasion Initiated by microRNA-106a

**DOI:** 10.1371/annotation/cbf27742-b6c5-4e1a-a7a1-c8141415b2d8

**Published:** 2012-08-21

**Authors:** Bo Feng, Tao Tao Dong, Miang Liang Wang, Lin Lin Wang, Hou Min Zhou, Hong Chao Zhao, Feng Dong, Min Hua Zheng

Due to an error in the production process, Ming Liang Wang was not included in the author byline. He should be listed as the third author, and his affiliations are 1 Department of Surgery, Ruijin Hospital, Shanghai Jiaotong University School of Medicine, Shanghai, People’s Republic of China, 2 Shanghai Institute of Digestive Surgery, Shanghai, People’s Republic of China, 3 Shanghai Minimally Invasive Surgery Center, Shanghai, People’s Republic of China. The contributions of this author are as follows: Conceived and designed the experiments, performed the experiments, analyzed the data, contributed reagents/materials/analysis tools, and wrote the manuscript.

The correct citation is: Feng B, Dong TT, Wang ML, Wang LL, Zhou HM, et al. (2012) Colorectal Cancer Migration and Invasion Initiated by microRNA-106a. PLoS ONE 7(8): e43452. doi:10.1371/journal.pone.0043452 

