# Supplementary material for: Colorectal Cancer Migration and Invasion Initiated by microRNA-106a
Source: PLoS One. 2012 Aug 17;7(8):e43452. doi: 10.1371/journal.pone.0043452 (PMC3422256; doi:10.1371/journal.pone.0043452)
Supplement: Table S1 — Correlation of miR-106a expression with metastasis in colorectal cancer patients. (DOC) [file pone.0043452.s001.doc]

| Table S1 Correlation of miR-106a expression with metastasis in colorectal cancer patients | | | | | | | | |
| --- | --- | --- | --- | --- | --- | --- | --- | --- |
| No. | sex | localisation | TNM-status | Grading | R-Classif. | metastasis at diagnosis | metastasis in follow-up | miR-106a expression |
| 1 | f | left side | pT3pN1pM1 | 3 | 0 | yes |  | higher |
| 2 | f | left side | pT3pN1pM1 | 3 | 0 | yes |  | lower |
| 3 | f | left side | pT3pN1pM1 | 3 | 0 | yes |  | higher |
| 4 | f | left side | pT3pN1pM0 | 3 | 0 |  | yes | higher |
| 5 | f | right side | pT3pN0pM0 | 2 | 0 |  | yes | higher |
| 6 | f | right side | pT3pN1pM0 | 2 | 0 |  | yes | higher |
| 7 | f | right side | pT3pN1pM0 | 2 | 0 |  | yes | lower |
| 8 | m | right side | pT3pN0pM0 | 2 | 0 |  | yes | higher |
| 9 | f | right side | pT3pN1pM0 | 2 | 0 |  | yes | higher |
| 10 | m | left side | pT3pN0pM0 | 2 | 0 |  | yes | lower |
| 11 | f | right side | pT3pN0pM0 | 2 | 0 |  | yes | higher |
| 12 | m | left side | pT3pN1pM0 | 2 | 0 |  | yes | higher |
| 13 | f | right side | pT3pN1pM0 | 2 | 0 |  | yes | lower |
| 14 | f | left side | pT3pN1pM0 | 3 | 0 |  | yes | higher |
| 15 | m | right side | pT3pN1pM0 | 2 | 0 |  | yes | higher |
| 16 | f | left side | pT3pN1pM0 | 2 | 0 |  | yes | higher |
| 17 | f | left side | pT3pN1pM0 | 2 | 0 |  | yes | higher |
| 18 | f | left side | pT3pN1M0 | 2 | 0 |  | yes | lower |
| 19 | f | right side | pT3pN1M0 | 2 | 0 |  | yes | higher |
| 20 | m | left side | pT3pN1pM0 | 3 | 0 |  | yes | lower |
| 21 | f | right side | pT3pN1pM0 | 3 | 0 |  | yes | higher |
| 22 | m | right side | pT3pN1pM0 | 2 | 0 |  | yes | higher |
| 23 | m | right side | pT3pN1pM0 | 2 | 0 |  | no | higher |
| 24 | m | right side | pT3pN1pM0 | 2 | 0 |  | no | higher |
| 25 | f | right side | pT3pN1pM0 | 3 | 0 |  | no | lower |
| 26 | f | right side | pT3pN1pM0 | 3 | 0 |  | no | lower |
| 27 | f | left side | pT3pN1pM0 | 3 | 0 |  | no | higher |
| 28 | f | right side | pT3pN1pM0 | 3 | 0 |  | no | higher |
| 29 | f | left side | pT3pN1pM0 | 3 | 0 |  | no | lower |
| 30 | m | left side | pT3pN1pM0 | 3 | 0 |  | no | lower |
| 31 | m | right side | pT3pN1pM0 | 3 | 0 |  | no | higher |
| 32 | m | right side | pT3pN1pM0 | 3 | 0 |  | no | lower |
| 33 | f | left side | pT3pN1pM0 | 3 | 0 |  | no | lower |
| 34 | f | left side | pT3pN1pM0 | 3 | 0 |  | no | lower |
| 35 | f | right side | pT3pN1pM0 | 3 | 0 |  | no | higher |
| 36 | f | left side | pT3pN1pM0 | 3 | 0 |  | no | lower |
| 37 | f | left side | pT3pN1pM0 | 3 | 0 |  | no | lower |
| 38 | m | right side | pT3pN1pM0 | 3 | 0 |  | no | lower |
| 39 | f | right side | pT3pN1pM0 | 3 | 0 |  | no | higher |
| 40 | f | left side | pT3pN1pM0 | 3 | 0 |  | no | lower |
| 41 | m | left side | pT4pN0M0 | 2 | 0 |  | no | lower |
| 42 | f | right side | pT3pN1pM0 | 2 | 0 |  | no | higher |
| 43 | m | right side | pT3pN1M0 | 2 | 0 |  | no | lower |
| 44 | m | right side | pT3pN1pM0 | 2 | 0 |  | no | higher |
| 45 | m | right side | pT3pN1pM0 | 2 | 0 |  | no | lower |
| 46 | m | right side | pT3pN1pM0 | 2 | 0 |  | no | higher |
| 47 | m | left side | pT3pN1M0 | 2 | 0 |  | no | higher |
| 48 | m | right side | pT3pN1M0 | 2 | 0 |  | no | lower |
| 49 | m | right side | pT3pN1M0 | 2 | 0 |  | no | lower |
| 50 | m | right side | pT3pN1M0 | 2 | 0 |  | no | higher |
